# Microbiology of wetlands

**DOI:** 10.3389/fmicb.2013.00079

**Published:** 2013-04-05

**Authors:** Paul L. E. Bodelier, Svetlana N. Dedysh

**Affiliations:** ^1^Department of Microbial Ecology, Netherlands Institute of Ecology (NIOO-KNAW)Wageningen, Netherlands; ^2^Winogradsky Institute of Microbiology, Russian Academy of SciencesMoscow, Russia

## Introduction

Wetlands are ecologically as well as economically important systems due to their high productivity, their nutrient (re)cycling capacities, and their prominent contribution to global greenhouse gas emissions. Being on the transition between terrestrial and—aquatic ecosystems, wetlands are buffers for terrestrial run off thereby preventing eutrophication of inland as well as coastal waters. The close proximity of oxic–anoxic conditions, often created by wetland plant roots, facilitates the simultaneous activity of aerobic as well as anaerobic microbial communities. Input of nutrients and fast recycling due to active aerobes and anaerobes makes these systems highly productive and therefore attractive for humans as well as many other organisms. Wetlands globally are under high pressure due to anthropogenic activities as well as climate change. Changes of land-use as well as altered hydrology due to climate change will lead to disturbance and loss of these habitats. However, the diversity and functioning of microbial communities in wetland systems is highly underexplored in comparison to soils and aquatic ecosystems.

The special issue in *Frontiers in Terrestrial Microbiology* offers the collection of 4 review articles and 14 original research papers, which contribute to the current knowledge on the microbiology of wetlands and discuss the gaps therein to be assessed in future wetland research. Notably, these studies address a wide variety of wetland types including rice paddies (Alam and Jia, 2012; Conrad et al., 2012), acidic *Sphagnum*-dominated peatlands (Bragina et al., 2011; Ivanova and Dedysh, 2012; Kolb and Horn, 2012; Preston et al., 2012; Putkinen et al., 2012; Sun et al., 2012), riparian wetlands (Wang et al., 2012), black mangroves (Laanbroek et al., 2012), superficial aquatic sediments (Gu et al., 2012), salt marshes (Irvine et al., 2012; Lovell and Davis, 2012), and littoral boreal wetlands (Siljanen et al., 2012).

## Key-developments in wetland microbiology

The key developments in wetland microbiology of the first decade of the twenty-first century are summarized in a series of four reviews (Kolb and Horn, 2012; Lamers et al., 2012; Lovell and Davis, 2012; Pester et al., 2012). The most general overview of the microbially-driven conversions in wetlands is given by Lamers et al. (2012), who analyze the effect of microbial activities on growth and performance of plants. As conclusively shown in this paper, many biogeochemical conversions catalyzed by microbes can ultimately control vegetation composition in wetlands. This is especially the case for conversions in nitrogen, sulfur, and iron cycling. The authors present and propose research strategies and priorities to integrate understanding of plant-microbial interactions in wetlands. Three other reviews focus on several microbial functional guilds such as sulfate reducers (Pester et al., 2012) diazotrophs (Lovell and Davis, 2012), methanotrophs and denitrifiers (Kolb and Horn, 2012). As argued by Pester et al. (2012), although forming a small population, sulfate reducers in freshwater wetlands are capable of catalyzing substantial sulfate reduction rates and interacting significantly with microbes involved in other cycles. In contrast to common association of sulfate reduction with marine habitats, freshwater wetlands harbor a highly diverse sulfate-reducing community, largely comprised of microbes not related to cultured representatives. An important role of nitrogen fixing diazotrophs in the maintenance of nutrient limited salt marshes is highlighted by Lovell and Davis (2012), who demonstrate that the highly diverse diazotrophic community displays clear biogeography within the salt marsh and even differs between plant species, pointing at niche differentiation of diazotrophs within wetlands. Finally, a mini review by Kolb and Horn (2012) focuses on methanotrophs and denitrifiers that consume atmospheric greenhouse gases CH_4_ and N_2_O in acidic wetlands. One part of this review sheds more light on the potential of the indigenous methanotrophs to consume atmospheric CH_4_ (≤1.75 ppmv), a fact that is currently underappreciated for acidic northern wetlands. The other part summarizes the emergent evidence for the relevance of N_2_O consumption in acidic wetlands and reviews the current knowledge on acid-tolerant denitrifiers.

In line with the lack of knowledge on the microbiology of wetland habitats, most research papers either focus on characterizing microbial communities in terms of composition in relation to environmental factors or link important biogeochemical reactions to the responsible microbial communities.

## Microbial abundance, diversity, and spatial distribution

The study of Preston et al. (2012) applied multiple approaches to characterize depth-dependent microbial community structure and function in two geographically separated ombrotrophic bogs and a minerotrophic fen within the James Bay Lowlands, a large peatland complex of northern Ontario, Canada. Archaeal, bacterial, and fungal community structures in these three peatlands were characterized with terminal restriction fragment length polymorphism of ribosomal DNA and the microbial activity was measured using community level physiological profiling, extracellular enzyme activities, and the carbon mineralization rates of various natural and synthetic substrates. Despite differences in nutrient content, similar dominant microbial taxa were observed at all three peatlands. In contrast, basal respiration, enzyme activity, and the magnitude of substrate utilization were generally higher at a minerotrophic fen and similar between the two bogs. As concluded by the authors, microbial activity in peatlands appears to be determined by the quality of the peat substrate and the presence of potential microbial inhibitors.

A more detailed analysis of the bacterial communities colonizing two phylogenetically related *Sphagnum* mosses, *S. fallax* and *S. angustifolium*, is offered in a study by Bragina et al. (2011). Despite a distinct habitat preference of these two mosses with respect to the nutrient level, the hyaline cells of their leaves were dominated by members of the *Alphaproteobacteria*. Diversity of this numerically abundant bacterial group and the nitrogen-fixing prokaryotes associated with *Sphagnum* species was further investigated by a barcoded pyrosequencing approach, which confirmed high similarity of the microbial assemblages on plantlets of both mosses. The extensive survey of the *nifH* gene diversity presented in this study is the first pyrosequencing-based insight into diversity of nitrogen-fixing prokaryotes in *Sphagnum*-dominated wetlands and, therefore, is of particular interest.

Apart of the nitrogen-fixing *Alphaproteobacteria*, planctomycetes represent another group of bacteria that are typical for acidic northern wetlands. As shown by Ivanova and Dedysh (2012), members of the *Planctomycetes* comprise up to 14% of total bacterial cells detected by fluorescence *in situ* hybridization in acidic peat. *Planctomycetes* inhabit both oxic and anoxic peat layers and are highly diverse, although most of these bacteria belong to as-yet-uncultivated taxa. Based on the currently available knowledge, *Planctomycetes* have been proposed to play a role of slow-acting decomposers of plant-derived organic matter in northern wetlands, which are crucial ecosystems in global carbon cycling.

The three abovementioned articles on acidic *Sphagnum*-dominated wetlands (Bragina et al., 2011; Ivanova and Dedysh, 2012; Preston et al., 2012) are followed by the study of Wang et al. (2012), who focus on assessing the spatial patterns of iron- and methane-oxidizing bacterial communities in a circum-neutral, irregularly flooded, riparian wetland. This work addresses an intriguing question about the competition for limiting amounts of oxygen between iron- and methane-oxidizing aerobic bacteria. Special attention in this study is given to *Gallionella*-related iron oxidizers, since the authors developed a number of molecular assays to specifically detect and enumerate these bacteria. The results show that *Gallionella*-related iron oxidizers are highly abundant in the studied wetland and outnumber various groups of methane-oxidizers, demonstrating that iron-oxidizers are both able to compete for oxygen with methanotrophs as well as with chemical iron oxidation.

Finally, the paper of Laanbroek et al. (2012) introduces the Black mangrove wetlands as a microbial habitat to the reader. The specific focus in this study is on diversity of ammonia-oxidizing *Betaproteobacteria*, which reflects the changes in hydrology in three different Black mangrove habitats, i.e., locations with dwarf, sparse, and dense trees. *Nitrosomonas*- and *Nitrosospira*-related bacteria were identified as the major ammonia-oxidizers in these mangroves. Small, but significant differences in the bacterial communities between the flooded and non-flooded impoundments were also detected.

## Biogeochemical transformations driven by microbes in wetlands

A long standing question in methane emission from rice paddies is to what extent rice straw affects the pathway of methane formation. Using a comprehensive combination of stable isotope fractionation and molecular detection techniques Conrad et al. (2012) demonstrated that pathways of methane formation in degradation of different types of straw (rice vs. maize) were rather similar despite the involvement of differentially composed methanogenic communities in the soils used. Hence, the temporal patterns and path of methane formation was mainly controlled by the soil type rather than by type of straw. The methane formation process was stable despite the fluctuating composition of the communities involved. In turn, the fluctuating communities may be related to the pathway of carbon degradation and the resulting substrates for methane formation.

The study of Sun (Sun et al., 2012) examined the methane production rates in relation to the diversity and dynamics of methanogens in three peatlands with contrasting characteristics: two acidic peat bogs and a minerotrophic fen. The focus in this study was on analyzing inducible shifts in methanogen populations in response to substrates (acetate and hydrogen) added to peat in short-term incubation experiments. The acetate-amendment stimulated rates of CH_4_ production in a fen peatland soil and increased the relative abundance of the *Methanosarcinaceae*. By contrast, addition of H_2_ stimulated CH_4_ production in two acidic bog soils and enhanced abundance of the E2 group of the *Methanoregulaceae*. As concluded by the authors, variation in the supply of metabolic substrates is a driving force of methanogen species-sorting in wetlands. Hence, methane formation and emission from wetland soils are predominantly controlled by methanogenic substrates.

Next to substrates for energy generation methanogens also need elements for building up biomass, a fact which has never been looked at in wetland systems. Irvine et al. (2012) demonstrate that methanogens in salt marshes can be N-limited, which is an alternative explanation for enhanced methane emission from wetlands upon N-additions. This possibility was not considered as generally increased methane emissions upon addition of nitrogen were assumed to be caused by both increase in plant biomass and inhibition of methane consumption. This finding urges to re-thinking of nitrogen control of methane emission from wetlands and opens up many possibilities for new research.

The consumption of methane in wetlands and upland soils has been shown to be influenced by nitrogen and nitrogenous fertilizers, however, solid mechanistic explanations substantiated by experimental data are still lacking. Alam and co-workers (Alam and Jia, 2012) executed incubation studies using rice soil and demonstrated that addition of nitrogenous fertilizers to certain levels stimulated specific methane oxidizers (i.e., type I) which confirms what has been demonstrated before for many different rice soils. However, higher doses of ammonium-based fertilizers inhibited methane oxidation which may act through the activity of nitrifiers given the strong negative correlation between methane oxidation and nitrate production, designating the importance of interaction between microbial groups for methane emission from rice paddies.

In contrast, applying nitrogen *in situ* to a natural littoral wetland in a boreal lake had neither an effect on methane oxidation potential nor on methane flux. In the study by Siljanen et al. (2012) nitrogen load activated *pmoA* gene transcription of type I methanotrophs, but decreased the relative abundance of *pmoA* gene transcripts of type II methanotrophs, so that the overall methanotroph activity was not affected by the nitrogen amendment. Hence, the evaluation of the effect of nitrogen load on methane oxidation has to include laboratory as well as *in situ* observations and can differ significantly between different types of wetlands.

Besides nitrogen, the distribution and dispersion of methanotrophic bacteria can play a regulating role in methane cycling in wetland ecosystems as demonstrated by Putkinen et al. (2012). These authors addressed the role of water dispersal in colonization of *Sphagnum* mosses by methanotrophic bacteria. It is known that *Sphagnum* plantlets and in particular hyaline cells of these mosses are colonized by methanotrophs, which are responsible for oxidizing CH_4_ on its way from anoxic peat layers to the atmosphere. As shown in this study, inactive methanotroph-free *Sphagnum* plantlets were able to acquire methane-oxidizing activity and the respective methanotroph population after few days of transplantation next to methanotroph-containing mosses or after incubation in peat water taken from a methanotroph-active wetland site. This colonization was suggested as a resilience mechanism for peatland CH_4_ dynamics by allowing the re-emergence of methane oxidation activity in *Sphagnum*.

The last contributions to this special issue focus on interactions between elemental cycles (Fe-N, S-N cycle) in groundwater and wetland sediments. The possible presence of iron-oxidizing nitrate reducers and potentially co-occurring iron reducers was assessed in an iron-sulfide and nitrate rich groundwater in a freshwater wetland by Haaijer et al. (2012). Molecular analyses indicated a potential role of nitrate reducing iron-oxidizers. However, cultivation attempts only resulted in the isolation of *Geobacter*-like iron-reducers demonstrating that a polyphasic approach is required to get an unbiased picture of microbial elemental cycling and their interactions in wetlands systems. Interactions between sulfur and nitrogen cycles in a wide range of surface sediments, representing variation in major controlling factors were assessed by Gu et al. (2012). Using flow-through reactors nitrate- and sulfate-reducing rates were measured in the presence and absence of nitrate and sulfate. The multivariate analyses of N- and S cycling rates with the wide range of environmental conditions designated pH and salinity to be the most important regulating factors with little interference between nitrate and sulfate reduction. The observed coupling between nitrate reduction and sulfide oxidation may have implications for metal availability and toxicity in wetland systems.

## Synthesis

The research compiled in this special issue on wetland microbiology clearly demonstrates that our understanding of the microbes living in wetland ecosystems is far from complete. The eminent role of wetlands for humanity combined with the crucial role microbes play in the functioning of these threatened ecosystems necessitates the more intensive and comprehensive assessment of microbiology of wetlands in the near future research. Significant changes in the hydrology of wetlands are predicted as a result of climate change, hence deeper knowledge of wetlands as a component of terrestrial ecosystems must be achieved. However, the complexity of wetlands and the microbial ecosystems therein makes these systems unamendable for a straightforward approach. More knowledge is needed at all levels going from single-cell ecophysiology to *in situ* biogeochemical functioning. Hence, persistent cultivation efforts and combined stable isotope-genomic approaches in combination with appropriate environmental physico-chemical characterization are required to achieve a more profound understanding of microbes and microbial communities in wetlands.
